# Association between ergonomic risk exposures and insomnia symptoms: a mediation analysis of the 5th Korean working conditions survey

**DOI:** 10.1186/s12889-024-17659-y

**Published:** 2024-01-10

**Authors:** Seong-Sik Cho, Tae-Won Jang, Mo-Yeol Kang

**Affiliations:** 1 Department of Occupational and Environmental Medicine, College of Medicine, Dong-A University of Korea, Busan, Republic of Korea; 2 Department of Occupational and Environmental Medicine, College of Medicine, Hanyang University, Seoul, Republic of Korea; 3 Department of Occupational and Environmental Medicine, Seoul St. Mary’s Hospital, College of Medicine, The Catholic University of Korea, 222, Banpo-daero, Seocho-gu, 06591 Seoul, Republic of Korea

**Keywords:** Insomnia symptoms, Occupational exposure, Ergonomic risks, Korean Working conditions Survey

## Abstract

**Background:**

This study investigates the relationship between ergonomic risk exposures and insomnia symptoms, using data representative of Korea’s general working population.

**Methods:**

Data from the 5th Korean Working Conditions Survey were used for this study. The eligible population (employees) for the current study was 37,026. Insomnia symptoms were estimated using the minimal insomnia symptom scale (MISS) questionnaire. Logistic regression analysis was conducted to explore the association between ergonomic risks and insomnia symptoms.

**Results:**

All the investigated ergonomic risks increased odd ratios (ORs) for insomnia symptoms: Tiring or painful positions (OR, 1.64; 95% CI, 1.43–1.88); lifting or moving heavy loads (OR, 2.33; 95% CI, 1.99–2.71); long periods of standing (OR, 1.47; 95% CI, 1.29–1.69); and repetitive hand or arm movements (OR, 1.46; 95% CI, 1.29–1.67). The mediated proportion of musculoskeletal pain was 7.4% (95% CI, 5.81–10.13), and the mediated proportion of feeling of exhaustion was 17.5% (95% CI, 5.81–10.13).

**Conclusions:**

This study provides evidence for the relationship between ergonomic risks and insomnia symptoms, for which musculoskeletal pains and the feeling of exhaustion may be potential mediators.

**Supplementary Information:**

The online version contains supplementary material available at 10.1186/s12889-024-17659-y.

## Background

Sleep issues are often quite common among working population [1]. Approximately 8% of the working population suffers from insomnia, and about 30% and about 30% displays symptoms of it. Insomnia is associated with many health problems, especially cardiovascular disease [2], type 2 diabetes [3], hypertension [4], obesity [5], and mental disorders [6]. Moreover, insufficient or poor quality of sleep leads to various symptoms such as fatigue [7], decreased cognitive performance [8], and impaired recovery from physical health losses [9]. This, in turn, may affect work performance and productivity as well as increase the risk of accidents and injuries at work, sickness absence, and work disability [10, 11].

Significant proportions of workers have to perform strenuous tasks characterized by awkward postures and heavy lifting, often without sufficient rest periods, which could lead to an overload response [12], increasing their risk for sleep problems [13]. Some cross-sectional studies associate ergonomic risk exposures with sleep problems and insomnia. Among 3,727 working registered nurses in the USA, disturbed sleep was associated with repeated and monotonous movements, twisted physical postures, breaking into a sweat every day, shaking and vibrating, and moving or lifting heavy loads [14]. Likewise, long walks at work, lifting and/or heavy manual labor, as well as prolonged periods of intense physical exertion at work, also caused poor sleep in a large cohort of Australian women [15]. However, other studies reported contradictory results, observing that ergonomic risk exposures are not significantly associated with poor sleep quality, shorter sleep durations, and insomnia. For instance, a prospective Swedish study with a two-year follow-up found that awkward work positions, heavy lifting, or supranormal physical exertion did not increase the risk of sleep problems [16], which has also previously been reported [17]. These inconsistent results invite further exploration of the association between work demands and insomnia in other working populations.

Therefore, this study investigated the association between ergonomic risk exposures and insomnia symptoms, using data representing Korea’s general working population. Furthermore, we explored potential mediators between ergonomic risk exposures and insomnia symptoms., such as musculoskeletal pain and feeling of exhaustion.

## Methods

### Study sampling and participants

The current study analyzed data from the 5th Korean Working Conditions Survey (KWCS), which the Korean Occupational Safety and Health Agency conducted. The survey aimed to assess the Korean labor force’s comprehensive working conditions, safety, and health. The KWCS is comparable to the European Working Conditions Survey or the British Labour Force Survey in terms of content and structure.

The KWCS employed a three-stage probability proportion stratified cluster sample design. First, census districts were selected using a systematic sampling method based on probability proportional to size to reflect the number of households in each census district. Second, systematic sampling was used to select ten households from each selected census district randomly. One interviewee was chosen randomly from each eligible household (eligible individuals participated in the labor market at the time of the survey). The survey was conducted between July and November of 2017. Face-to-face interviews were conducted by trained interviewers. Survey weighting was estimated by adjusting the sampling design, the non-response rate, and the post-stratification to ensure that the survey was representative of Korean working populations. The external and content validity and reliability was assured in previous study [18].

The population of the KWCS comprises all workers aged over 15 years. The current study analyzed data from employees who participated in the KWCS. The study did not analyze employers, self-employed workers, unpaid family workers, and other non-employees. The total study population for the 5th KWCS was 50,176, whereas the eligible population (employees) for the current study was 37,026.

### Ergonomic risk exposures

Exposures to ergonomic risks were determined by multiplying exposure scales and weekly working hours. If participants were exposed to ergonomic risk factors for at least 20 h per week, they were considered as exposed to ergonomic risks. In contrast, if their weekly exposure duration was less than 20 h, they were not considered exposed to ergonomic risk. The combination of the question assessed each exposure to ergonomic risk “does your main paid job involve…?” and the following descriptors: “Tiring or painful positions,” “Carrying or moving heavy loads,” “Long periods of standing,” and “Repetitive hand or arm movements.” Exposure scales were classified as “always” (1.0), “nearly always” (0.95), “approximately three-fourths of the time” (0.75), “approximately one-half of the time” (0.5), “approximately one-fourth of the time” (0.25), “almost never” (0.05), and “never” (0).

### Insomnia symptoms

The minimum insomnia symptom scale (MISS) questionnaire examined insomnia symptoms. The MISS questionnaire contains three components: “difficulty in initiating sleep,” “difficulty maintaining sleep,” and “non-restorative sleep.” Participants mentioned the frequency of each sleep-related symptom using the following descriptors: “every day” (four points), “sometimes a week” (three points), “occasionally in a month” (two points), “rarely” (one point), “not at all” (zero points), and “don’t know” and “refusal” (both of which were considered as non-responses). MISS scores ranged from 0 to 12. According to an assessment study on the validity of MISS, it exhibits good measurement properties as an insomnia-screening questionnaire and cut-off point of ≥ 6 allows for valid comparisons [19, 20]. Hence, scores of 0–5 are classified as the absence of insomnia symptoms, whereas scores of 6 or higher indicate insomnia symptoms.

### Mediators

**Fig. 1:**
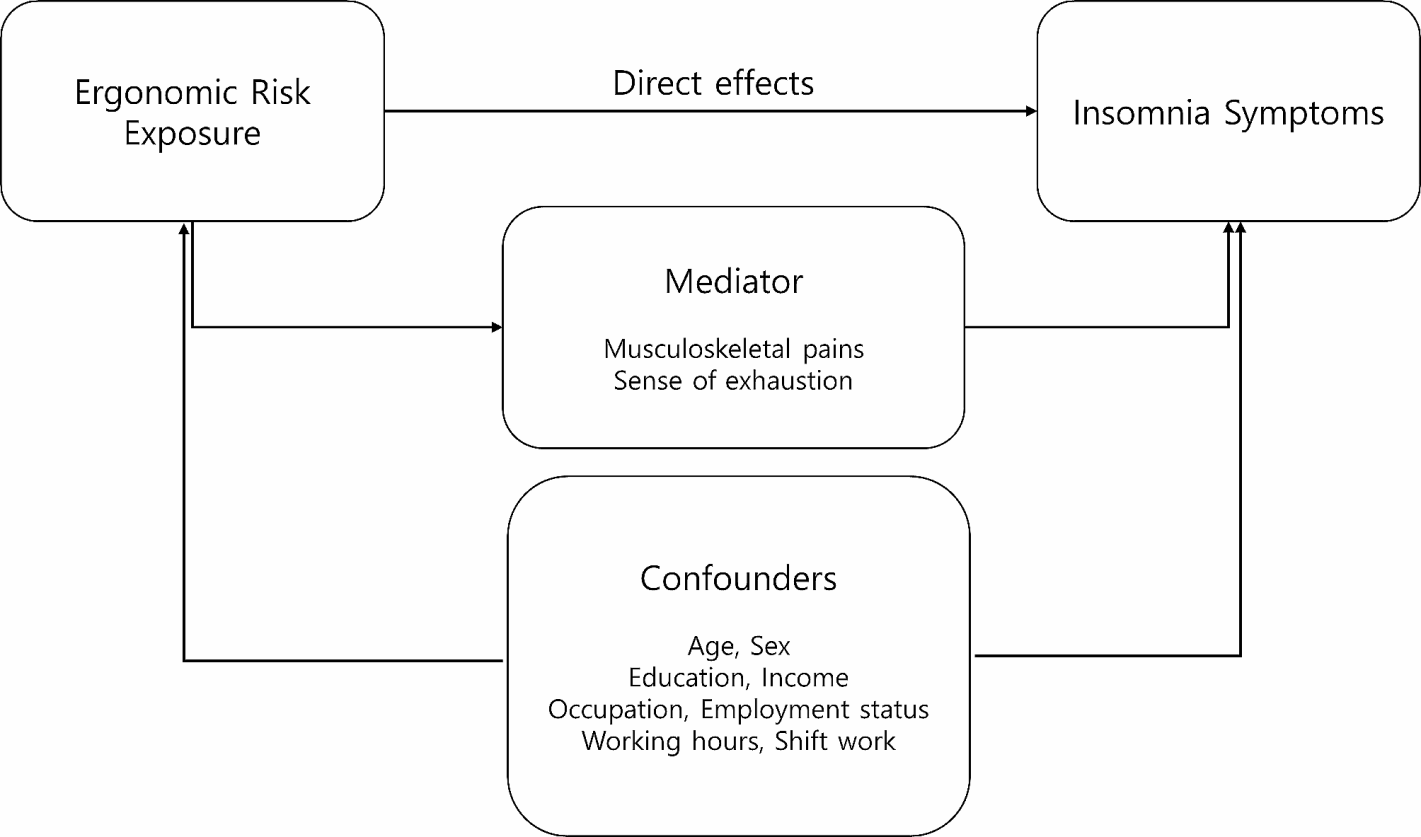
Conceptual diagram for the hypothesis that musculoskeletal pains and the feeling of exhaustion could be potential mediators between ergonomic risks and insomnia symptoms

We hypothesize that musculoskeletal pains and the feeling of exhaustion could be potential mediators between ergonomic risk factors and insomnia symptoms. Figure 1 illustrates the conceptual diagram for this hypothesis. Ergonomic risks can lead to musculoskeletal pain and mental exhaustion which are correlated with insomnia [21, 22]. The presence of musculoskeletal pains was estimated based on health problems experienced in the last 12 months. Respondents’ experience of backache, shoulder pain, neck pain, upper limb pain, or lower limb pain (hips, legs, knees, feet) constituted musculoskeletal pains. “Yes” or “NO” response was used for assessment of presence or absence of musculoskeletal pain. The feeling of exhaustion was assessed by a question about the frequency of “feeling of exhaustion at the end of the working day.” “Always,” “most of the time,” and ”sometimes” were considered to indicate the presence of a feeling of exhaustion. “Rarely” and “never” were regarded as the absence of the feeling of exhaustion.

### Social and occupational characteristics

Sex, age, and socioeconomic status (such as educational level, income, occupation, and employment status) were considered covariates. Moreover, weekly working hours, shift work, and health status (musculoskeletal pain and self-rated health) were considered. Age was classified into four age groups: 16–29, 30–39, 40–49, 50–59, and 60 years or older. The education level was divided into three groups: college graduate or higher education, high school graduate, and middle school graduate or lower education. The income level was divided into quartiles: lowest, lower-middle, upper-middle, and highest. Occupation was divided into four categories. The first group was management/professionals; the second was clerical workers; the third was service or sales workers, and the fourth was manual workers. A simple “yes” or “no” question (“Do you work shifts?”) was used to assess shiftwork. Working hours were classified into five groups based on their weekly working hours (1–34, 35–40, 41–5, 53–60, and 61 h or more).

### Statistical analysis

The numbers and percentages of employees across demographic and occupational characteristics were provided. The prevalence of insomnia symptoms was calculated for each ergonomic risk exposure. To investigate the association between ergonomic risk exposures and insomnia symptoms, odds ratios (ORs) and 95% confidence intervals (95% CIs) were calculated using survey-weighted logistic regression analysis. The results of the unadjusted model were shown; the adjusted model 1 included age, gender, and socioeconomic status (including educational level, income, occupations, and employment status); the adjusted model 2 additionally included weekly working hours and shift work; the fully adjusted model 3 additionally included musculoskeletal pains and the feeling of exhaustion to the previously included variables in model 2. Mediation analysis was conducted to estimate percentages of mediated effects. “Medeff,” which is users developed the Stata command, was utilized for mediation analysis [23]. “Medeff” is developed for causal mediation analysis and it can adjust potential confounders, basically it is based on the multivariable regression analysis. In the analysis, age, sex, education, income, occupation, employment status, weekly working hours, and shift work were adjusted in the model. Finally, subgroup analyses and interaction analyses were conducted based on the social and occupational factors. The Stata Ver 17 was used for all statistical analyses (Stata Co., College Station, Texas, USA).

## Results

Table 1. demonstrates the distributions of ergonomic risks across the social and occupational characteristics. A higher proportion of men (70.2%) were exposed to ergonomic risks. The highest proportion of individuals (77.5%) with high school graduate degree were exposed to ergonomic risks. Regarding occupation and employment status, the manual workers (79.6%) and those who had daily employment (71.6%) were more frequently involved in ergonomic risk exposures. Moreover, musculoskeletal pain and the feeling of exhaustion were linked to higher levels of ergonomic risk exposures.

**Table 1:** Characteristics of the study population

	Total		Ergonomic risk (-)		Ergonomic risk (+)	
	n	percent*	n	percent**	n	percent**
Gender						
Male	21,022	56.8	6261	29.8	14,761	70.2
Female	16,004	43.2	5010	31.3	10,994	68.7
Age group						
16–39	16,135	43.6	4825	29.9	11,310	30.1
40–49	9364	25.3	2894	30.9	6470	69.1
50–59	7424	20.0	2002	27.0	5422	73.0
60+	4103	11.1	1550	37.8	2553	62.2
Education						
Middle school or lower	3197	8.6	1148	35.9	2049	64.1
High school	11,525	31.2	2595	22.5	8930	77.5
College or higher	22,273	60.2	7508	33.7	14,765	66.3
Income						
Lowest	7265	20.9	2985	39.9	4370	60.1
Lower-middle	8129	23.4	1976	24.3	6153	75.7
Upper-middle	9447	27.2	2351	24.9	7096	75.1
Highest	9896	28.5	3365	34.0	6531	66.0
Employment						
Regular	30,001	81.0	8727	29.1	21,273	70.9
Temporary	5109	13.8	1999	39.1	3110	60.9
Daily	1917	5.2	544	28.4	1373	71.6
Occupation						
Professional & managerial	8792	23.8	2897	33.0	5895	67.0
Clerical	9042	24.5	3854	42.6	5195	57.4
Sales & service	7449	20.2	2112	28.4	5337	71.6
Manual	11,618	31.5	2370	20.4	9248	79.6
Weekly working hours						
1–34	3851	10.4	2502	65.0	1349	35.0
35–40	18,172	49.1	5798	31.9	12,374	68.1
41–52	10,146	27.4	2207	22.7	7838	77.3
53–60	3647	9.9	502	13.8	3145	86.2
61+	1209	3.3	161	13.3	1048	86.7
Shift work						
No	32,641	88.2	10,325	31.6	22,316	68.4
Yes	4381	11.8	944	21.6	3437	78.4
Musculoskeletal pain						
No	28,448	76.8	9224	33.8	18,824	66.2
Yes	8574	23.2	1645	19.2	6929	80.8
Feeling of exhaustion at the end of the working day						
No	28,983	78.3	9495	32.4	19,579	67.6
Yes	8016	21.7	1856	23.1	6160	76.9

*: column precent, **: row percent, ergonomic risk (+): exposure to at least one ergonomic risk factors (tiring or painful positions, lifting or moving people, lifting heavy loads, standing for long duration, and repetitive hand or arm movements), *p*-value is calculated by chi-square test

Table 2. shows the prevalence of insomnia symptoms according to their ergonomic risk exposures. All categories of investigated ergonomic exposures increased the prevalence of insomnia symptoms. Workers who were not exposed to ergonomic risk factors had a prevalence of 4.8%, while workers exposed to at least one ergonomic risk had a prevalence of 7.5%. Notably, the prevalence of insomnia symptoms was the highest among workers involved in heavy loads (12.9%) and the second highest among workers with tiring or painful positions (9.8%). Furthermore, with the increase in the number of exposures, the prevalence increased gradually from 4.8 to 16%.

**Table 2:** Ergonomic risk exposures and prevalences of insomnia symptoms

	Total		Sleep disturbance (-)		Sleep disturbance (+)		
	n	percent*	n	percent**	n	percent**	*p*
Tiring or painful positions							< 0.001
(-)	26,979	72.9	25,479	94.4	1500	5.6	
(+)	10,041	27.1	9058	90.2	983	9.8	
Lifting heavy loads							< 0.001
(-)	32,024	86.5	30,188	94.3	1836	5.7	
(+)	4991	13.5	4346	87.1	645	12.9	
Standing for a long duration							< 0.001
(-)	21,951	59.3	20,722	94.4	1230	5.6	
(+)	15,064	40.7	13,812	91.7	1252	8.3	
Repetitive hand or arm movements							< 0.001
(-)	17,055	46.1	16,146	94.7	909	5.3	
(+)	19,961	53.9	18,388	92.1	1573	7.9	
At least one ergonomic risk exposure							< 0.001
(-)	11,261	30.4	10,723	95.2	538	4.8	
(+)	25,753	69.6	23,809	92.5	1944	7.5	
Numbers of ergonomic risk exposure							< 0.001
0	11,261	30.4	10,723	95.2	538	4.8	
1	11,058	29.9	10,430	94.3	628	5.7	
2	7555	20.4	7040	93.2	515	6.8	
3	4650	12.6	4249	91.4	401	8.6	
4	2481	6.7	20,854	84.0	396.0	16.0	

*: column percent, **: row percent, *p*-value is calculated by chi-square test

Table 3. displays the connection between ergonomic risk exposures and insomnia symptoms using survey-weighted logistic regression analysis. All investigated ergonomic risk exposures increased odds ratios for insomnia symptoms. In the fully adjusted model (model 3), the following exposures were associated with insomnia symptoms: Tiring or painful positions (OR, 1.64; 95% CI, 1.43–1.88); lifting or moving heavy loads (OR, 2.33; 95% CI, 1.99–2.71); long periods of standing (OR, 1.47; 95% CI, 1.29–1.69); repetitive hand or arm movements (OR, 1.46; 95% CI, 1.29–1.67) and at least one physical demand (OR, 1.60; 95% CI, 1.37–1.86). Also, as the number of exposures increased, the odds ratios increased from one ergonomic exposure: 1.31 (95%CI: 1.09–1.56) to four ergonomic exposures: 3.86 (95%CI: 3.07–4.85).

**Table 3:** Association between ergonomic risks and insomnia symptoms according to survey-weighted logistic regression analysis

	Unadjusted			Model 1			Model 2			Model 3		
Risk factors	OR	95%CI		OR	95%CI		OR	95%CI		OR	95%CI	
Tiring or painful positions	1.84	1.65	2.06	1.85	1.63	2.09	1.85	1.63	2.10	1.64	1.43	1.88
Lifting heavy loads	2.44	2.15	2.77	2.66	2.29	3.08	2.67	2.30	3.10	2.33	1.99	2.71
Standing for a long duration	1.53	1.37	1.71	1.58	1.39	1.81	1.60	1.39	1.83	1.47	1.29	1.69
Repetitive hand or arm movements	1.52	1.36	1.71	1.56	1.38	1.77	1.60	1.41	1.82	1.46	1.29	1.67
At least one physical demand	1.63	1.42	1.86	1.70	1.47	1.97	1.77	1.52	2.05	1.60	1.37	1.86
**Number of exposed risk factors**	**OR**	**95%CI**		**OR**	**95%CI**		**OR**	**95%CI**		**OR**	**95%CI**	
0	reference			reference			reference			reference		
1	1.20	1.02	1.41	1.28	1.07	1.52	1.35	1.13	1.62	1.31	1.09	1.56
2	1.46	1.23	1.73	1.63	1.35	1.97	1.72	1.42	2.09	1.54	1.27	1.87
3	1.88	1.57	2.24	2.11	1.72	2.57	2.24	1.82	2.75	1.95	1.58	2.41
4	3.79	3.16	4.55	4.51	3.65	5.57	4.85	3.91	6.02	3.86	3.07	4.85

Model 1: age, sex, education, income, occupation, and employment status were adjusted

Model 2: age, sex, education, income, occupation, employment status, working hours, and shift work were adjusted

Model 3: age, sex, education, income, occupation, employment status, working hours, shift work, musculoskeletal pain, and the sense of exhaustion were adjusted

Table 4. provides the estimated percentages of effects mediated by mediation analysis (Medeff). In this analysis, musculoskeletal pain and the feeling of exhaustion were hypothesized as mediators. From ergonomic risk exposures to insomnia symptoms, smaller percentages were mediated by musculoskeletal pains (the range of mediated percentage: 2.18–6.36), while more significant proportions were mediated by the feeling of exhaustion (the range of mediated percentage: 12.0–16.6). It indicates that musculoskeletal pains and the feeling of exhaustion partially explain insomnia symptoms associated with ergonomic risk exposures.

**Table 4:** Mediated percent of musculoskeletal pains and feeling of exhaustion on insomnia symptoms according to mediation analysis

	Musculoskeletal pain*			Feeling of exhaustion *		
	Percent	95%CI		Percent	95%CI	
Tiring or painful positions	4.37	3.70	5.21	12.96	11.05	15.41
Lifting heavy loads	2.18	1.86	2.54	12.02	10.34	13.92
Standing for a long duration	4.47	3.47	6.19	16.08	12.53	22.40
Repetitive hand or arm movements	6.46	5.10	8.94	16.62	13.11	24.02
At least one ergonomic risk exposure	7.44	5.81	10.13	17.45	13.65	23.34

Mediated proportions were estimated by the medeff(a Stata command developed by users); *: age, sex, education, income, occupation, employment status, weekly working hours, and shift work were adjusted in the model.

Table 5. shows the association between exposure to ergonomic risk exposures and insomnia symptoms across various subgroups. Interactions with ergonomic exposure were seen in educational and income level, employment status, musculoskeletal pain. However, ergonomic risk factors increased ORs for insomnia symptoms in most subgroups. Regarding age group, employees in their fifties were most vulnerable to insomnia symptoms when exposed to ergonomic risks. Regarding employment status and occupation, regular workers showed the highest odds ratio (OR, 1.76; 95% CI, 1.52–2.07), and service and sales workers were more susceptible to insomnia symptoms. Sales and service workers’ OR for ergonomic risk were found to be the highest (OR, 2.26; 95% CI, 1.73–2.94). Regarding shiftwork, shift workers’ OR for ergonomic risk was statistically insignificant. Interestingly, musculoskeletal pain increased insomnia symptoms regardless of their exposure to ergonomic risk.

**Table 5:** Association between at least one ergonomic risk exposure and insomnia symptoms across different subgroups

	Ergonomic risk exposure	Total	Sleep disturbance (-)	Sleep disturbance (+)	OR (95% CI)	P for interaction
		n(percent)*	n(percent)**	n(percent)**		
Sex						
Male	(-)	6258(29.8)	5986(95.7)	272(4.3)	ref	
	(+)	14,760(70.2)	13,701(92.8)	1059(7.2)	1.70(1.39–2.07)	
Female	(-)	5003(31.3)	4736(94.7)	267(5.3)	ref	
	(+)	10,993(68.7)	10,108(91.9)	885(8.1)	1.56(1.30–1.86)	0.51
Age						
16–39	(-)	4820(29.9)	4622(95.9)	198(4.1)	ref	
	(+)	111,310(70.1)	10,517(93.0)	793(7.0)	1.76(1.38–2.24)	
40–49	(-)	2890(30.9)	2742(94.9)	148(5.1)	ref	
	(+)	6469(69.1)	5993(92.6)	476(7.4)	1.47(1.14–1.91)	0.325
50–59	(-)	2002(27.0)	1904(95.1)	98(4.9)	ref	
	(+)	5422(73.0)	4952(91.3)	470(8.7)	1.84(1.38–2.44)	0.824
≥ 60	(-)	1550(37.8)	1455(93.9)	95(6.1)	ref	
	(+)	2553(62.2)	2347(91.9)	206(8.1)	1.35(1.02–1.78)	0.156
Education						
Middle school or lower	(-)	1148(35.9)	1067(92.9)	81(7.1)	ref	
	(+)	2049(64.1)	1894(92.4)	155(7.6)	1.07(0.79–1.46)	
High school	(-)	1148(35.9)	1067(92.9)	81(7.1)	ref	
	(+)	2049(64.1)	1894(92.4)	155(7.5)	1.52(1.19–1.93)	0.082
College or higher	(-)	7498(33.7)	7181(95.8)	314(4.2)	ref	
	(+)	14,765(66.3)	13,699(92.8)	1066(7.2)	1.78(1.47–2.14)	0.007
Income						
Lowest	(-)	2893(39.8)	2720(94.0)	173(6.0)	ref	
	(+)	4369(60.2)	4042(92.5)	327(7.5)	1.27(0.99–1.62)	
Lower-middle	(-)	1976(24.3)	1865(94.4)	111(5.6)	ref	
	(+)	6153(75.7)	5690(92.5))	463(7.5)	1.36(1.04–1.79)	0.691
Upper-middle	(-)	2348(24.9)	2276(96.9)	72(3.1)	ref	
	(+)	7095(75.1)	6620(93.3)	475(6.7)	2.27(1.64–3.16)	0.006
Highest	(-)	3364(34.0)	3246(96.5)	118(3.5)	ref	
	(+)	6531(66.0)	6032(92.4)	499(7.6)	2.26(1.66–3.10)	0.005
Employment status						
Regular	(-)	8720(29.1)	8333(95.6)	387(4.4)	ref	
	(+)	21,272(70.9)	19,657(92.4)	1615(7.6)	1.76(1.52–2.07)	
Temporary	(-)	1997(39.1)	1881(92.1)	116(5.8)	ref	
	(+)	3109(60.9)	2864(92.1)	245(7.9)	1.39(1.01–1.90)	0.173
Daily	(-)	544(28.4)	509(93.4)	36(6.6)	ref	
	(+)	1372(71.6)	1288(93.9)	84(6.1)	0.93(0.56–1.55)	0.018
Occupation						
Professional & managerial	(-)	2891(32.9)	2761(95.5)	130(4.5)	ref	
	(+)	5895(67.1)	5478(92.9)	417(7.1)	1.61(1.20–2.17)	
Clerical (office work)	(-)	3851(42.6)	3682(95.6)	169(4.4)	ref	
	(+)	5195(57.4)	4820(92.8)	375(7.2)	1.70(1.28–2.24)	0.081
Sales & service	(-)	2112(28.4)	2024(95.8)	88(4.2)	ref	
	(+)	5336(71.6)	4861(91.1)	475(8.9)	2.26(1.73–2.94)	0.099
Manual	(-)	2370(20.4)	2221(93.7)	149(6.3)	ref	
	(+)	9247(79.6)	8576(92.7)	671(7.3)	1.16(0.93–1.47)	0.097
Weekly working hours						
1–34	(-)	2500(64.9)	2337(93.5)	163(6.5)	ref	
	(+)	1349(35.1)	1242(92.1)	107(7.9)	1.23(0.89–1.70)	
35–40	(-)	5791(31.9)	5535(95.6)	256(4.4)	ref	
	(+)	12,373(68.1)	11,577(93.6)	796(6.4)	1.49(1.21–1.82)	0.328
41–52	(-)	2307(22.7)	2227(96.5)	80(3.5)	ref	
	(+)	7838(77.3)	7191(91.7)	647(8.3)	2.51(1.85–3.40)	0.002
53–60	(-)	499(13.7)	476(95.3)	23(4.7)	ref	
	(+)	3148(86.3)	2879(91.4)	269(8.6)	1.90(1.00-3.64)	0.235
≥ 61	(-)	207(16.0)	188(90.9)	19(9.1)	ref	
	(+)	1092(84.0)	964(88.2)	128(11.8)	1.17(0.55–2.48)	0.902
Shift work						
No	(-)	10,315(31.6)	9847(95.5)	468(4.5)	ref	
	(+)	22,314(68.4)	20,690(92.7)	1624(7.3)	1.65(1.43–1.90)	
Yes	(-)	944(21.6)	873(92.5)	71(7.5)	ref	
	(+)	3437(78.4)	3116(90.6)	321(9.4)	1.27(0.84–1.90)	0.224
Musculoskeletal pain						
No	(-)	9221(33.8)	7881(96.0)	327(4.0)	ref	
	(+)	18,823(66.2)	17,479(92.9)	1344(7.1)	1.78(1.51–2.08)	
Yes	(-)	1638(19.1)	1500(91.5)	67(8.5)	ref	
	(+)	6928(80.9)	6328(91.3)	600(8.7)	1.02(0.79–1.34)	0.001
Feeling of exhaustion						
No	(-)	5382(38.5)	5251(97.6)	131(2.4)	ref	
	(+)	8597(61.5)	8303(96.6)	294(3.4)	1.54(1.31–1.80)	
Yes	(-)	5868(25.5)	4513(93.8)	297(6.1)	ref	
	(+)	17,140(74.5)	15,490(90.4)	1650(9.6)	1.42(1.11–1.83)	0.967

*: column percent; **:row percent

Appendix table 1 shows co-exposure of musculoskeletal pain and ergonomic risk on insomnia symptoms and appendix table 2 shows co-exposure of feeling of exhaustion and ergonomic risk on insomnia symptoms. In the both tables, infra-additive interactions were observed.

## Discussion

Considering the high prevalence of sleep disorders and their health consequences among the working population, identifying the risk factors for poor sleep and sleep problems is important and would help devise strategies to promote a healthier, safer, and more productive workforce. Previous studies suggest that insomnia is related to gender, levels of education or socioeconomic status, marital status, smoking and alcohol consumption, caffeine intake, and psychiatric comorbidities, such as depression and anxiety [24–26]. Another important area of interest for sleep is the work environment. A range of workplace factors have the potential to influence employees’ sleep, including shift work, psychosocial stress, and physical work environment (e.g., noise and extreme temperature), as well as exposure to chemical and infectious agents. Among these, psychosocial factors have been emphasized in various studies, but the potential effects of physically demanding tasks or ergonomic risks on sleep remain relatively neglected.

This study aimed to investigate the association between ergonomic risk exposures and insomnia symptoms in a nationwide representative sample of the Korean working population. The findings suggest that ergonomic risk exposures are associated with insomnia symptoms. Even in stratified analysis by age, sex, education, income level, occupation, employment type, weekly working hours, shift work musculoskeletal pain, and the feeling of exhaustion, the ergonomic risks retained a statistically significant relationship with insomnia symptoms. Therefore, we could conclude that these workplace ergonomic risk factors (physically demanding work) unfavorably contributed to the workers’ sleep health.

Our findings are consistent with results of previous research that work-related physical burden has a negative effect on sleep, unlike leisure-time physical activity. In a study analyzing the U.S. Hispanic population, the amount of activity in each domain of physical activity was examined according to sleep time [27]. As a result, there was no significant difference in the transportation and leisure domains, but in the work-related domain, sleep time and physical activity amount showed an inversely proportional pattern. In other words, people who slept relatively little were mainly people who engaged in a lot of work-related physical activity, and people who slept a lot were people who had little work-related physical activity. In a study conducted in Russia, also similar to our findings, heavy physical work was found to cause difficulty in falling asleep [28]. Likewise, the results of a cross-sectional analysis of the Danish PHysical ACTivity cohort data suggested that occupational physical activity was associated with the risk of insomnia, and, as in our study, a clear dose-response relationship was observed [29]. When analyzing Korean firefighters, the risk of insomnia increased in groups with high subjective or objective occupational physical activity, while the risk is significantly lower among those with leisure-time physical activity [30].

Various social, psychological, and physical factors—including chronic musculoskeletal pain, physical fatigue, poor working environment, job stress, and additional occupational hazards—might mediate the relationship between high physical demands at work and insomnia. We attempted to understand whether the ergonomic risks and potential mediators, such as musculoskeletal pain and feeling of exhaustion, influence insomnia symptoms. The results showed that feelings of exhaustion and musculoskeletal pain mediate around 17% and 7% of the relationship between ergonomic risks and insomnia symptoms, respectively. These factors partially explain the relationship between ergonomic risk exposures and insomnia symptoms. This was consistent with previous research, which showed that chronic musculoskeletal pain, fatigue, and poor health are potential mediators for a negative association between work-related ergonomic risk exposures and the risk of insomnia [22, 31]. Another possible explanation is that poor sleep conditions may affect perceptions of the working environment, which could not determine causal directions in the current cross-sectional context, as our research utilized subjective measures. Individuals with poor sleep due to other factors (e.g., poor health, loneliness, isolation, unfavorable family environment, lack of sleep) may be more emotionally responsive, which can affect their subjective assessment of their work situation [16, 32].

The strengths of the current study include using a nationally representative survey of the working population, an assessment of insomnia symptoms using a validated tool, and the available information on several potential confounders that have seldom been explored in literature. Despite the strengths, however, the study has the following limitations. First, as the study design was cross-sectional, a causal relationship could not be verified. Furthermore, given the healthy worker effect, we could not exclude the possibility of workers with sleep problems changing jobs or leaving the labor market because of their occupational physical burden, which may underestimate the true associations. Second, information on insomnia symptoms and exposures was obtained using self-administered questionnaires; this method relied on the accuracy of the responder’s memory and may be subject to non-response bias and recall bias. Specifically, although we consider weekly working hours, ergonomic risk exposures were defined and categorized operationally with unstructured questions. For example, even with the same ergonomic factor, the load on the musculoskeletal system will differ depending on the quantitative and qualitative loads. Hence, our assessment method only provides contextual information, not quantitative data. Moreover, measurement of mediators, musculoskeletal pains and the feeling of exhaustion, was relied on single questions. Third, this study particularly evaluated relationship between ergonomic risk exposures and insomnia symptoms without considering key confounding factors such as sleep medication use, history of mental illness (e.g., major depressive disorder, anxiety disorder), other health behaviors (e.g., smoking, alcohol drinking, leisure-time physical activity), because the information provided in the survey did not include these variables.

In conclusion, the current study provides evidence for the relationship between ergonomic risk exposures and insomnia symptoms among the general working population in South Korea. In future studies, overcoming the limitations of our study, a causal relationship should be confirmed using a longitudinal design and an objective assessment method and by gathering detailed information about exposure and confounders. Moreover, future studies should investigate the interplay between both the domains of physical and psychosocial work demands and the risk of sleep problems, considering the complexity and the interaction of the latent factors and mediators in the relationship.

### Electronic supplementary material

Below is the link to the electronic supplementary material.

**Appendix Table 1**. Joint influence of musculoskeletal pains and ergonomic risks on insomnia symptoms. **Appendix Table 2**. Joint influence of feeling of exhaustion and ergonomic risk on insomnia symptoms.

## Data Availability

The datasets analyzed during the current study are available on the Korean Working Conditions Survey Homepage, available at https://oshri.kosha.or.kr/eoshri/resources/KWCSDownload.do.

## Abbreviations

CI: Confidence Interval
KWCS: Korean Working Conditions Survey
MISS: Minimal Insomnia Symptom Scale
OR: Odd Ratio
