# Supplementary material for: Association between ergonomic risk exposures and insomnia symptoms: a mediation analysis of the 5th Korean working conditions survey
Source: BMC Public Health. 2024 Jan 10;24:149. doi: 10.1186/s12889-024-17659-y (PMC10782752; doi:10.1186/s12889-024-17659-y)
Supplement: Supplementary file 1 — Appendix Table 1. Joint influence of musculoskeletal pains and ergonomic risks on insomnia symptoms. Appendix Table 2. Joint influence of feeling of exhaustion and ergonomic risk on insomnia symptoms. [file 12889_2024_17659_MOESM1_ESM.docx]

| **Appendix Table 1. Joint influence of musculoskeletal pains and ergonomic risks on insomnia symptoms** | | | | | | |  |
| --- | --- | --- | --- | --- | --- | --- | --- |
|  | **Musculoskeletal pain (-)** | | | **Musculoskeletal pain (+)** | | |  |
|  | **Insomnia symptoms (-)** | **Insomnia symptoms (+)** | **OR*(95%CI)** | **Insomnia symptoms (-)** | **Insomnia symptoms (+)** | **OR*(95%CI)** |  |
|  | n(percent) | n(percent) |  | n(percent) | n(percent) |  | p for interaction |
| Tiring or painful positions (-) | 20959(95.1) | 1091(4.9) | ref | 3678(92.7) | 292(7.3) | 1.37(1.13-1.67) |  |
| Tiring or painful positions (+) | 5795(89.8) | 656(10.2) | 1.93(1.66-2.25) | 4182(90.3) | 447(9.7) | 1.77(1.48-2.11) | p<0.001 |
| Lifting heavy loads (-) | 23914(94.8) | 1306(5.2) | ref | 5954(92.5) | 484(7.5) | 1.34(1.14-1.59) |  |
| Lifting heavy loads (+) | 2841(86.6) | 440(13.4) | 2.75(2.31-3.28) | 1908(88.2) | 255(11.8) | 2.37(1.92-2.92) | p<0.001 |
| Standing for a long duration (-) | 15879(95.2) | 807(4.8) | ref | 3357(92.2) | 283(7.8) | 1.52(1.23-1.88) |  |
| Standing for a long duration (+) | 10878(92.0) | 941(8.0) | 1.68(1.44-1.98) | 4498(91.0) | 456(9.0) | 1.78(1.47-2.16) | p<0.001 |
| Repetitive hand or arm movements (-) | 12549(95.6) | 584(4.4) | ref | 2252(91.8) | 201(8.2) | 1.73(135-2.21) |  |
| Repetitive hand or arm movements (+) | 14206(92.4) | 1164(7.6) | 1.73(1.49-2.02) | 5607(91.2) | 538(8.8) | 1.78(1.49-2.15) | p<0.001 |
| Exposure to at least one ergonomic risk factor (-) | 7851(96.0) | 327(4.0) | ref | 959(93.4) | 67(6.6) | 1.44(0.96-2.14) |  |
| Exposure to at least one ergonomic risk factor (+) | 18874(93.0) | 1422(7.0) | 1.72(1.42-2.09) | 6899(91.1) | 672(8.9) | 2.01(1.62-2.50) | 0.001 |
| *: adjusted by age, sex, education, income, occupation, employment status, weekly working hours, shift work, and feeling of exhaustion; OR (odds ratio); CI (confidence Interval) | | | | | | | |

| **Appendix Table 2. Joint influence of feeling of exhaustion and ergonomic risk on insomnia symptoms** | | | | | | |  |
| --- | --- | --- | --- | --- | --- | --- | --- |
|  | **Feeling of exhaustion (-)** | | | **Feeling of exhaustion (+)** | | |  |
|  | **Insomnia symptoms (-)** | **Insomnia symptoms (+)** | **OR*(95%CI)** | **Insomnia symptoms (-)** | **Insomnia symptoms (+)** | **OR*(95%CI)** |  |
|  | n(percent) | n(percent) |  | n(percent) | n(percent) |  | p for interaction |
| Tiring or painful positions (-) | 10843(97.4) | 293(2.6) | ref | 13779(92.7) | 1091(7.3) | 2.96(2.42-3.62) |  |
| Tiring or painful positions (+) | 2734(95.4) | 132(4.6) | 1.83(1.36-2.47) | 7234(88.2) | 971(11.8) | 4.99(4.04-6.18) | 0.524 |
| Lifting heavy loads (-) | 12360(97.3) | 340(2.6) | ref | 17487(92.3) | 1451(7.7) | 2.96(2.47-3.55) |  |
| Lifting heavy loads (+) | 1217(93.5) | 85(6.5) | 2.69(1.90-3.81) | 3524(85.2) | 610(14.8) | 6.77(5.47-8.36) | 0.004 |
| Standing for a long duration (-) | 8464(97.4) | 227(2.6) | ref | 10757(92.6) | 863(7.4) | 2.88(2.28-3.64) |  |
| Standing for a long duration (+) | 5110(96.3) | 198(3.7) | 1.45(1.09-1.93) | 10253(89.6) | 1198(10.5) | 4.40(3.47-5.58) | 0.245 |
| Repetitive hand or arm movements (-) | 6565(97.8) | 149(2.2) | ref | 8224(92.8) | 636(7.2) | 3.26(2.49-4.25) |  |
| Repetitive hand or arm movements (+) | 7011(96.2) | 276(3.8) | 1.71(1.27-2.30) | 12784(90.0) | 1425(10.0) | 4.81(3.74-6.20) | 0.133 |
| Exposure to at least one ergonomic risk factor (-) | 4316(97.8) | 98(2.2) | ref | 4513(93.8) | 297(6.2) | 2.72(1.91-3.89) |  |
| Exposure to at least one ergonomic risk factor (+) | 9258(96.6) | 328(3.4) | 1.54(1.08-2.20) | 164498(90.3) | 1765(9.7) | 4.65(3.36-6.44) | 0.398 |
| *: adjusted by age, sex, education, income, occupation, employment status, weekly working hours, shift-work, and musculoskeletal pain; OR(Odds ratio); CI(confidence Interval) | | | | | | | |
